# Environment mediated multipartite and multidimensional entanglement

**DOI:** 10.1038/s41598-019-45496-2

**Published:** 2019-06-24

**Authors:** Chee Kong Lee, Mojdeh S. Najafabadi, Daniel Schumayer, Leong Chuan Kwek, David A. W. Hutchinson

**Affiliations:** 1 Centre for Quantum Technologies, National University of Singapore, Singapore, 117543 Singapore; 2 Department of Chemistry, Massachusetts Institute of Technology, Cambridge, Massachusetts 02139 USA; 3 Dodd-Walls Centre for Photonic and Quantum Technologies, Department of Physics, University of Otago, Dunedin, New Zealand; 4 Institute of Advanced Studies, Nanyang Technological University, 60 Nanyang View, Singapore, 639673 Singapore; 5 National Institute of Education, 1 Nanyang Walk, Singapore, 637616 Singapore; 6 MajuLab, CNRS-UNS-NUS-NTU International Joint Research Unit, Singapore, UMI 3654 Singapore; 7 Department of Physics, Clarendon Laboratory, University of Oxford, Parks Road, Oxford, OXI 3PU United Kingdom

**Keywords:** Ultracold gases, Quantum information, Qubits

## Abstract

Quantum entanglement is usually considered a fragile quantity and decoherence through coupling to an external environment, such as a thermal reservoir, can quickly destroy the entanglement resource. This doesn't have to be the case and the environment can be engineered to assist in the formation of entanglement. We investigate a system of qubits and higher dimensional spins interacting only through their mutual coupling to a reservoir. We explore the entanglement of multipartite and multidimensional system as mediated by the bath and show that at low temperatures and intermediate coupling strengths multipartite entanglement may form between qubits and between higher spins, i.e., qudits. We characterise the multipartite entanglement using an entanglement witness based upon the structure factor and demonstrate its validity versus the directly calculated entanglement of formation, suggesting possible experiments for its measure.

## Introduction

Entanglement between systems with few degrees of freedom is often a fragile property as their interaction with a much larger environment drives decoherence. This decoherence often comes from the small system interacting in an incoherent, memory-less manner with the bath. Most theoretical studies of few-body quantum systems make this assumption explicitly or implicitly, i.e., the dynamics is Markovian. Such approximation seems quite appropriate and experimentally valid to high precision in condensed matter systems^1^. However, it is also a possibility that the environment, if suitably prepared, can mediate the creation of quantum correlation between small systems^2^.

Quantum entanglement for two or even three qubits has been studied extensively^3–8^ and it is well understood, but less well characterised is *multipartite entanglement* – entanglement between several systems. For example, for more than four qubits, the creation as well as the characterisation of quantum entanglement becomes exceedingly difficult. Theoretical investigations also face the curse of dimensionality, since the dimension of the corresponding Hilbert-space grows exponentially with the number of subsystems. Despite the hurdles, experimental demonstration of multipartite entanglement has been achieved in ion traps with up to 14 ions^9–11^, or for ten superconducting qubits^12,13^ and ten photonic qubits on a linear optical platform^14^.

The main obstacle to achieving significant multipartite entanglement is decoherence and dissipation due to coupling to the environment. The lifetime of entangled states decreases rapidly with the size of the system. Standard approaches to overcome this difficulty involve high fidelity quantum gates and large qubit overheads, which can be more and more challenging as the number of qubits increases. On the other hand, steady state two-qubit entanglement can be generated by engineered dissipation in trapped ions^15^ and superconducting qubits^16^, and noise assisted quantum transport has also been reported^17,18^. These experimental results challenge the general assumption that dissipation is always detrimental to quantum information processing, rather they demonstrate that it might be a resource that can be engineered and harnessed.

It has been conjectured that non-Markovian effects in the quantum time evolution, assuming an Ohmic bosonic bath at zero temperature, can leave their fingerprints on the time dependence of coherence^19^. This conjecture relies on the applicability and accuracy of the Born approximation underpinning the Bloch-Redfield equation. It should be noted that this perturbation approach contains accumulating divergences^20^, rendering the Born approximation inappropriate at long times. As a consequence care should be taken when interpreting these theoretical investigations, but the different Markovian and non-Markovian processes which affect relaxation and dephasing do offer opportunities to engineer the effects of the environment upon the system of interest.

Another research direction proposes entangling systems with more numerous degrees of freedom^21^. In general we may replace a standard two-level system with a *d*-dimensional system – a *qudit*. Such generalisation is not a simple extension of already existing concepts: the entanglement of two-qubit systems seems somewhat the exception^22^ rather than the rule. In addition, there is qualitative difference between bipartite and multipartite entanglement^23^ and the latter may provide higher flexibility to achieve more efficient quantum information processing^24^. The main focus of recent experiments has been to generate and characterise multilevel and multipartite entanglement^21,25–27^. Our work presented here aims to contribute to these efforts.

Here we build a theoretical model to generate steady state multipartite and multilevel entanglement mediated solely by the environment. First we consider a general open quantum system coupled to a bath and then we examine the equilibrium reduced density matrix of non-interacting qudits through the analytical polaron treatment, complemented by a numerically exact path-integral approach. Next, we demonstrate the use of the structure-factor-based and global entanglement witnesses to compute entanglement in bipartite and multilevel systems. For a non-interacting pair of qubits, we demonstrate that the entanglement is maximised at finite amount of system-bath interaction, and the peak values are temperature dependent.

Our results can be tested experimentally, as scattering experiments have already proved to be useful in quantifying multipartite entanglement^28^. Structure factors are macroscopic quantities measurable in various periodic systems, e.g., Bragg scattering on ultracold atoms in optical lattices^29^ or from ion chains^30^ and neutron scattering from solid state systems. Furthermore, the structure factor, particularly the dynamic structure factor, is a central observable quantity in determining single-particle and collective electronic excitations in many-body systems^31,32^.

## Model

**Figure 1:**
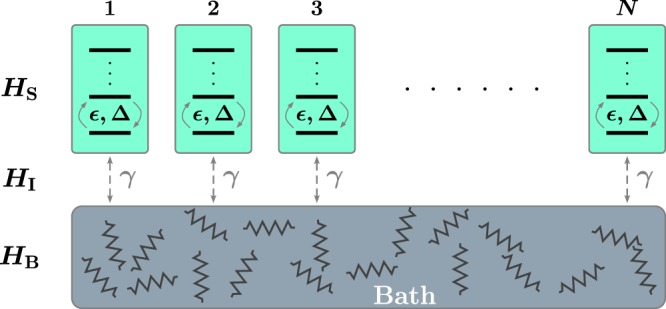
The schematics of the physical system consisting of *N d*-level systems (qudits) and a bath of harmonic oscillators. The qudits do not directly interact with each other, but they are all coupled to the bath.

We consider the collection of *N* identical, non-interacting qudits, see Fig. 1. The energy splittings between levels are assumed to be uniform and denoted by *ε* while the tunnelling matrix element is Δ. The bath is modelled as an ensemble of harmonic oscillators with frequencies *ω*_*n*_. The strength of coupling between a qudit and an oscillator with angular frequency *ω*_*n*_ is denoted by *g*_*n*_. Thus the total system-plus-bath Hamiltonian is formally written as *H*_tot_ = *H*_S_ + *H*_B_ + *H*_I_, where *H*_S_, and *H*_B_ govern the internal dynamics of the system and the bath, respectively, while *H*_I_ describes the coupling between them. In detail

1a $${H}_{{\rm{S}}}=\frac{1}{2}\sum _{j=1}^{N}\,[\epsilon {s}_{z}^{(j)}+\triangle {s}_{x}^{(j)}],$$

1b $${H}_{{\rm{B}}}=\sum _{n}\,{\omega }_{n}{b}_{n}^{\dagger }{b}_{n},$$

1c $${H}_{{\rm{I}}}=\sum _{n}\,\sum _{j=1}^{N}\,{g}_{n}{s}_{z}^{(j)}({b}_{n}^{\dagger }+{b}_{n}),$$

where *s*_*x*_ and *s*_*z*_ are the *x* and *z* components of a spin operator of a system with spin *s*, and the *b*_*n*_ are the bosonic bath operators. The number of degrees of freedom of the bath is assumed to be much larger than that of the qudits, thus according to the Poincaré recurrence theorem the time-scale on which the bath feeds back the energy to the subsystems is enormous.

This separation of time-scales allows us to smooth the oscillator modes and instead of {*ω*_*n*_} we characterise the bath with a continuous spectral density *J*(*ω*). For analytical calculations below, we assume a super-Ohmic spectrum, $J(\omega )=\gamma {(\frac{\omega }{{\omega }_{{\rm{c}}}})}^{3}{e}^{-\omega /{\omega }_{{\rm{c}}}}$, of exponential cut-off with a fixed *ω*_c_, and constant system-bath coupling strength, *γ*, with the dimension of frequency. The reciprocal of the cut-off frequency, $\tau \propto \frac{1}{{\omega }_{{\rm{c}}}}$ governs the relaxation time of the bath. This form of the spectral density is commonly used in the study of tunnelling effects in solid state systems^33^. Though the analysis below utilises a super-Ohmic spectrum, numerical results with other spectral densities, e.g., Ohmic and Lorentzian (see supplementary information), show that the conclusions are applicable to a wide range of systems. In order to capture the effects of strong qudit-bath coupling, we employ the polaron transformation defined as ${\tilde{H}}_{{\rm{tot}}}={e}^{P}{H}_{{\rm{tot}}}{e}^{-P}$, where

2 $$P=2\sum _{j}\,{s}_{z}^{(j)}\sum _{n}\,\frac{{g}_{n}}{{\omega }_{n}}({b}_{n}^{\dagger }-{b}_{n}).$$

The total Hamiltonian preserves its structure under this transformation: ${\tilde{H}}_{{\rm{tot}}}={\tilde{H}}_{{\rm{S}}}+{\tilde{H}}_{{\rm{B}}}+{\tilde{H}}_{{\rm{I}}}$, where ${\tilde{H}}_{{\rm{B}}}={H}_{{\rm{B}}}$ and

3a $${\tilde{H}}_{{\rm{S}}}=\frac{1}{2}\sum _{j=1}^{N}\,[\epsilon {s}_{z}^{(j)}+{{\rm{\Delta }}}_{{\rm{R}}}{s}_{x}^{(j)}]-\sum _{n}\,\frac{{g}_{n}^{2}}{{\omega }_{n}}\sum _{j,k}^{N}\,{s}_{z}^{(j)}{s}_{z}^{(k)}$$

3b $${\tilde{H}}_{{\rm{I}}}=\sum _{j=1}^{N}\,[{s}_{x}^{(j)}{V}_{x}+{s}_{y}^{(j)}{V}_{y}]$$

Several remarks are in order. First, the Hamiltonians are partitioned such that the expectation value ${\langle {\tilde{H}}_{{\rm{I}}}\rangle }_{{\rm{SB}}}={{\rm{Tr}}}_{{\rm{SB}}}({e}^{-\beta ({\tilde{H}}_{{\rm{S}}}+{\tilde{H}}_{{\rm{B}}})}{\tilde{H}}_{{\rm{I}}})=0$. Second, an effective qudit-qudit interaction term, $\sim {s}_{z}^{(j)}{s}_{z}^{(k)}$, appears in ${\tilde{H}}_{S}$, induced by the system-bath coupling. This term is the source of the finite equilibrium entanglement in the system since the qudits were originally assumed to be non-interacting. Third, the system-bath coupling in the polaron picture, ${\tilde{H}}_{{\rm{I}}}$, assumes a form very different from *H*_I_. All spin operators appear in a symmetric way and they are coupled to *effective bath* operators *V*_*x*_, *V*_*y*_. The explicit expressions of these bath operators are immaterial for our current analysis but for sake of transparency they are provided in the supplementary material. Finally, the polaron transformation renormalises the tunnelling rate, ${\rm{\Delta }}\mapsto {{\rm{\Delta }}}_{{\rm{R}}}$, and it thus picks up a temperature and coupling strength dependence.

The equilibrium reduced density matrix can formally be expressed^34^ as a partial trace

4 $${\tilde{\rho }}_{{\rm{S}}}=\frac{T{r}_{{\rm{B}}}({e}^{-\beta {\tilde{H}}_{{\rm{tot}}}})}{T{r}_{{\rm{SB}}}({e}^{-\beta {\tilde{H}}_{{\rm{tot}}}})}.$$

Expanding ${\tilde{\rho }}_{S}$ and taking into account that ${\langle {\tilde{H}}_{{\rm{I}}}\rangle }_{{\rm{SB}}}=0$ guarantees that in perturbative expansion the leading order term for ${\tilde{\rho }}_{{\rm{S}}}$ is of second order in the interaction Hamiltonian. The equilibrium state of the system can be approximated^35^ as ${\tilde{\rho }}_{{\rm{S}}}\approx {\tilde{\rho }}_{{\rm{S}},0}+{\tilde{\rho }}_{{\rm{S}},2}$ where

5a $${\tilde{\rho }}_{{\rm{S}},0}={Z}_{{\rm{S}},0}^{-1}\,{e}^{-\beta {\tilde{H}}_{{\rm{S}}}}$$

5b $${\tilde{\rho }}_{{\rm{S}},2}={Z}_{{\rm{S}},0}^{-2}[A{Z}_{{\rm{S}},0}-{Z}_{{\rm{S}},2}\,{e}^{-\beta {\tilde{H}}_{{\rm{S}}}}].$$

The leading ${\tilde{\rho }}_{{\rm{S}},0}$ term is the expected canonical equilibrium distribution in the polaron picture with ${Z}_{{\rm{S}},0}={{\rm{Tr}}}_{{\rm{S}}}({e}^{-\beta {\tilde{H}}_{{\rm{S}}}})$, while the correction term, ${\tilde{\rho }}_{{\rm{S}},2}$, picks up some of the correlation between the multi-level subsystems and the bath via

6 $$A=\sum _{\ell }\,{\int }_{0}^{\beta }\,d\tau {\int }_{0}^{\tau }\,d\tau ^{\prime} {C}_{\ell \ell }(\tau -\tau ^{\prime} ){e}^{-\beta {\tilde{H}}_{{\rm{S}}}}{S}_{\ell }(\tau ){S}_{\ell }(\tau \text{'}),$$

where ${S}_{\ell }=\sum _{j}\,{s}_{\ell }^{(j)}$ and *Z*_S,2_ = Tr_S_(*A*). Even the zeroth order term captures some correlation through the transformed system Hamiltonian. The exact expressions of ${C}_{\ell \ell }(\tau )={\langle {V}_{\ell }(\tau ){V}_{\ell }(\tau ^{\prime} )\rangle }_{{\tilde{H}}_{{\rm{B}}}}$ are in the supplementary information.

It is useful at this point to analyse the behaviour of perturbation theory at strong coupling in the polaron frame. As $\gamma \mapsto \infty$ the system becomes incoherent since the coherent tunnelling element, Δ_R_ vanishes. Simultaneously the correlation functions, ${C}_{\ell \ell }(\tau )$, and hence the second-order correction to the reduced density matrix ${\tilde{\rho }}_{{\rm{S}},2}$ vanishes. Therefore the equilibrium density matrix at strong *γ* is dominated by the bath induced coupling term, ${\sigma }_{z}^{(j)}{\sigma }_{z}^{(k)}$, e.g., for qubits ${\tilde{\rho }}_{{\rm{S}}}\propto \exp (\,-\,\beta {\tilde{H}}_{{\rm{S}}})$, where ${\tilde{H}}_{{\rm{S}}}$ is a diagonal matrix, since it is proportional to *σ*_*z*_. Therefore ${\tilde{\rho }}_{{\rm{S}}}$ is also diagonal. This result, namely that ${\tilde{\rho }}_{{\rm{S}}}$ follows the canonical Gibbs’ distribution, at least in the strong coupling regime, is in agreement with^36^. In the strong coupling (and limit of weak coupling) the density matrix can be factored as a product of single particle density matrices so this qubit system can be considered as a classical simulator in the language of  ^36^. It is only in the intermediate coupling regime, in the presence of entanglement, that the system provides a quantum resource.

## Entanglement Witness

The Hahn–Banach separation theorem guarantees that for any entangled state there is an entanglement witness. In the physics literature the concept of entanglement witness is predominantly defined as a functional, *W*, of the density matrix, *ρ*, such that Tr(*W*_*ρ*_) < 0 if *ρ* is entangled, and Tr(*W*_*ρ*_) > 0 if *ρ* is separable. However, currently there is no universal measure of entanglement for an arbitrary number of subsystems each possessing multiple levels, rather there are a plethora of functionals which are useful in specific situations. Unlike qudit and multipartite systems, bipartite qubit systems can be classified by a single measure^37^. This fact shows that bipartite qubit systems are not typical, rather the exceptions.

In order to quantify bipartite qubit entanglement, we adopt the standard measure of entanglement of formation (EoF)^38^. However, detecting multipartite entanglement for a mixed state is more complicated^7,39^, and we rely on the structure factor as a precursor of an entanglement witness

7 $${{\mathfrak{S}}}_{\ell \ell ^{\prime} }(k)=\sum _{i < j}\,{e}^{{\rm{i}}k({r}_{j}-{r}_{i})}\langle {s}_{\ell }^{(i)}{s}_{\ell ^{\prime} }^{(j)}\rangle ,$$

where $\ell$, $\ell ^{\prime}$ run over the set {*x*, *y*, *z*}, *k* is the wavenumber, and *r*_*i*_, *r*_*j*_ are the positions of spins *i* and *j*. The distance between two adjacent qubits is normalised to unity. The physical distance of two qubits is arbitrary. If the qubits are at equidistant locations with distance *a* then a structure factor ${{\mathfrak{S}}}_{\ell \ell ^{\prime} }(k,1)$ calculated with unit distance and another structure factor ${{\mathfrak{S}}}_{\ell \ell ^{\prime} }(k,a)$ calculated with a different distance, *a*, are related to each other as ${{\mathfrak{S}}}_{\ell \ell \text{'}}(k,1)={{\mathfrak{S}}}_{\ell \ell ^{\prime} }(\frac{k}{a},a)$. The entanglement witness, based on ${{\mathfrak{S}}}_{\ell \ell ^{\prime} }(k)$, is defined as *W*(*k*) = 1−Σ(*k*), where ${\rm{\Sigma }}(k)=\bar{{\rm{\Sigma }}}(k)+\bar{{\rm{\Sigma }}}(\,-\,k)$, and

8 $$\bar{{\rm{\Sigma }}}(k)=\frac{1}{2(\begin{array}{c}N\\ 2\end{array})}[{c}_{x}{{\mathfrak{S}}}_{xx}(k)+{c}_{y}{{\mathfrak{S}}}_{yy}(k)+{c}_{z}{{\mathfrak{S}}}_{zz}(k)].$$

It has been proven^39^ that *W* detects multipartite entanglement of a mixed state whenever *W* = *Tr*(*Wρ*) < 0, or equivalently 〈Σ〉 > 1. In general the coefficients ${c}_{\ell }$ can be chosen arbitrarily by the observer provided all ${c}_{\ell }$ are real and ${c}_{\ell }\le 1$. Below we use (*c*_*x*_, *c*_*y*_, *c*_*z*_) = (1, −1, 1). In the case of qudit systems we use a convenient lower bound^40^ of the entanglement of formation

9 $${\rm{EoF}}\ge {{\rm{EoF}}}_{{\rm{lb}}}=-\,{\mathrm{log}}_{2}(1-\frac{1}{2}{R}^{2}),$$

where

$$R=\frac{2}{\sqrt{C}}\sum _{\begin{array}{c}(j,k)\in C\\ j < k\end{array}}\,[|\langle j,j|\rho |k,k\rangle |-\sqrt{\langle j,k|\rho |j,k\rangle \langle k,j|\rho |k,j\rangle }],$$

with *C* being the set containing all pairs of indices (*j*, *k*), while |*C*| denotes its cardinality.

## Results

We first revisit the two-qubit case, i.e., ${s}_{\ell }\mapsto \frac{1}{2}{\sigma }_{\ell }$, and while this system has been analysed before^2,41^ we expand those analyses here. We use an imaginary time path-integral technique for calculating the reduced density matrix for the bare system, while for the dressed particles the previously described polaron transformation and second order approximation is employed since it yields accurate equilibrium density matrices for a wide range of parameters^35^, except for simultaneously low *ω*_*c*_ and temperatures.

In the weak coupling regime, increasing the bath mediated interaction between qubits enhances the induced entanglement. On the other hand, entanglement decreases exponentially as a function of *γ* in the strong coupling regime. The interaction term, $\sim {\sigma }_{z}^{(j)}{\sigma }_{z}^{(k)}$ in ${\tilde{H}}_{{\rm{S}}}$, dominates in the strong coupling limits, and the resulting ${\tilde{\rho }}_{{\rm{S}}}$ is localized (i.e., diagonal) in the *σ*_*z*_ basis with zero entanglement. The scaling limits the entanglement in both weak and strong coupling regimes and gives rise to the maximal entanglement at intermediate *γ*. The non-monotonic behavior of EoF as a function of *γ* may be reminiscent of the “stochastic resonance” phenomenon reported by Huelga^42^ and Lee^43^. Unlike the coupled quantum many-body systems considered in these earlier works, the qubits here are not coupled directly, the entanglement is solely induced by the interaction with the common reservoir.

It should be noted that ${\tilde{\rho }}_{{\rm{S}}}$ is quite different from *ρ*_S_. The transform describes a system “dressed” by the polaron cloud due to the system-bath interaction. However, this effective system is similar to those typically found in solid state quantum dots and BCS pairs. It is interesting to compare the amount of entanglement between two dressed qubits with that between two bare qubits. While the analytical polaron method is capable of exploring the entire range of dissipation strength and temperature, we need to employ the numerically exact imaginary time path-integral technique^44^ to obtain *ρ*_*S*_. Given the high computational cost of the path-integral technique, we restricted our analysis to small systems, at moderate *γ* and temperature values.

**Figure 2:**
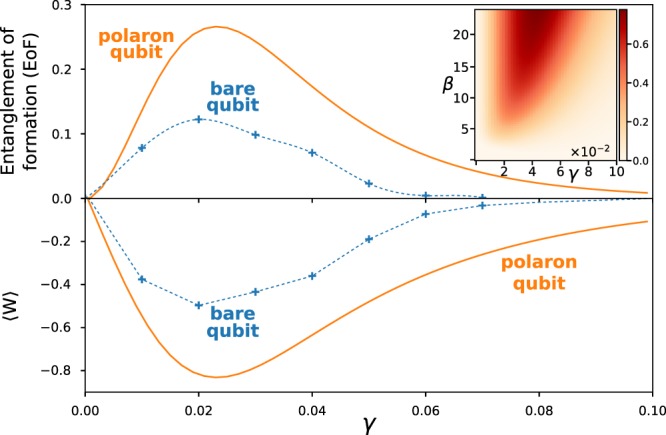
Two qubits: EoF and 〈*W*〉 are shown for two bare- and polaron qubits at *β* = 5. The dashed lines are only to guide the eye. Inset provides the EoF between two polaron qubits as a heatmap for varying interaction strength, *γ*, and inverse temperature, *β*.

The EoF between the qubits as a function of dissipation strength, *γ*, is shown in Fig. 2. The dependence of entanglement on *γ* between two bare qubits is similar to that of polaron qubits, albeit at a lower value. The difference of EoF between dressed qubits and bare qubits is a consequence of the system-phonon and phonon-phonon entanglement, captured through the polaron transformation but not in the calculation of entanglement between the bare qubits. The polaron transformation dresses the qubit with phonons in the bath and correlations in this phonon gas then contribute to the polaron entanglement. This result does not contradict our assumption of Markovian (memory less) time-evolution, rather it reflects the fact that within the environment equilibrium correlation has built up.

Figure 2 also illustrates the structure-factor-based entanglement witness, 〈*W*〉, for the two-qubit system. The negativity of 〈*W*〉 is proportional to the entanglement of formation. Of course 〈Σ〉 could also be used as an approximate measure of the entanglement. The advantage of both Σ and *W*, is that they are both directly related to the structure factor, ${{\mathfrak{S}}}_{\ell \ell ^{\prime} }(k)$ and one can thus connect to the wealth of knowledge in designing scattering experiments in order to interrogate the coupled system. Furthermore, adjusting the constants ${c}_{\ell }$’s one may explore different “pockets” of the high-dimensional Hilbert space^45^ and can further classify the entangled state.

The inset of Fig. 2 shows the EoF between two polaron qubits over a large range of *γ* and inverse temperature, *β*. It is observed that the bath mediated equilibrium entanglement is also sensitive to temperature, in addition to *γ*. At high temperature, all density matrices approach a totally mixed state since all eigenstates are equally populated, thus ${\tilde{\rho }}_{{\rm{S}}}\to 1$ and ${\tilde{\rho }}_{{\rm{S}},0}\to 1$ as *A* → 0. As a result, entanglement is destroyed at high temperatures, as expected. However, at lower temperatures the entanglement of dressed qubits develops and may become significant (~0.8).

**Figure 3:**
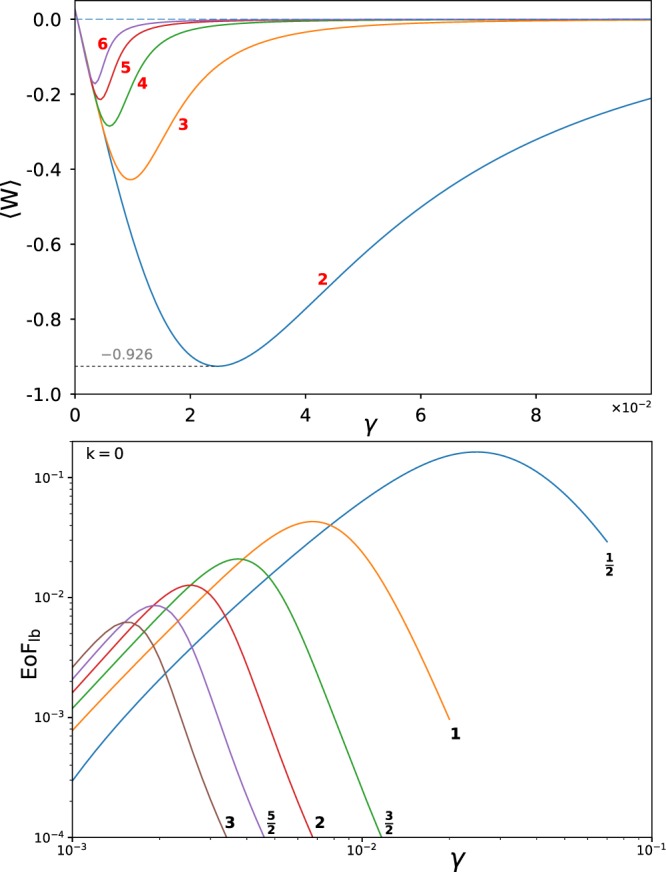
Entanglement witnesses, 〈*W*〉 for multipartite qubits for *N* = 2, 3, 4, 5, and 6 (main figure). Other parameter values are *β* = 5, *k* = 0, (*c*_*x*_, *c*_*y*_, *c*_*z*_) = (1, −1, 1), *ε* = 0, Δ = 1, and *ω*_*c*_ = 3. Bottom figure shows EoF_lb_ for bipartite qubits and qudits with total spin values from $s=\frac{1}{2}$, up to 3 in steps of $\frac{1}{2}$. Notice the log-log scale.

Following the illustrative two-qubit case, we next explore the possibility of observing bath induced multipartite entanglement. Let us consider a linear chain of up to six noninteracting qubits (*N* = 6) coupled to a common harmonic bath. Figure 3 demonstrates the behaviour of 〈*W*〉 for different system sizes, including *N* = 2–6 qubits (red bold labels). As seen, increasing the system size by one additional qubit causes a significant decrease in optimal peak of 〈*W*〉 from −0.926 to around −0.4 with the same noise level. Increasing the system size further, we again observe a lower optimum peak for 〈*W*〉. Measuring 〈*W*〉 for systems with 5 and 6 qubits shows that system size has a direct effect on the correlation and the maximal entanglement moves to lower ranges in the coupling for larger systems.

**Figure 4:**
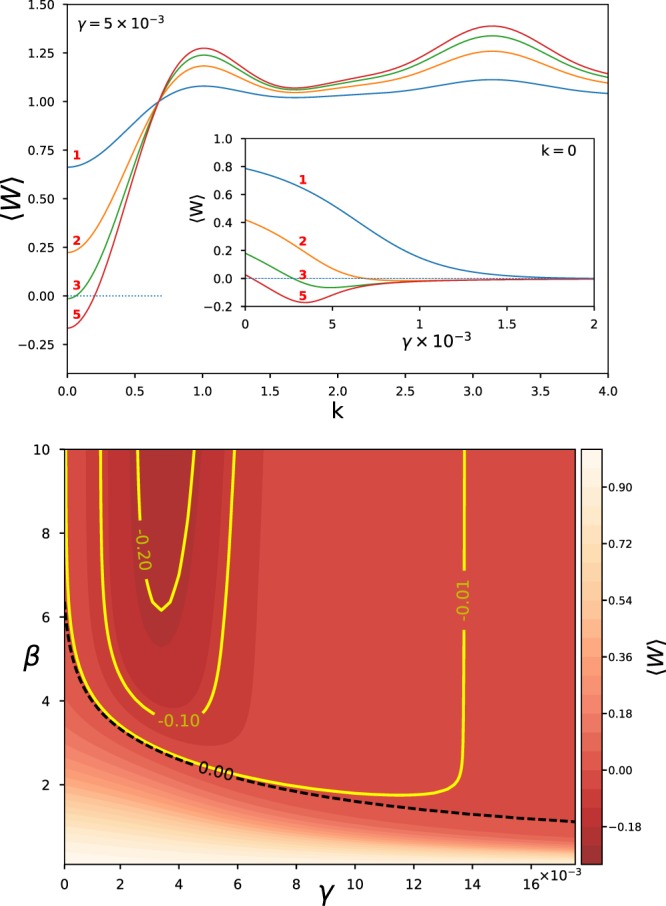
Six qubits. (Top) Entanglement witness 〈*W*〉 is plotted as a function of *k* with fixed interaction strength *γ* = 5 × 10^−3^. The inset depicts the dependence of 〈*W*〉 on *γ* for the fixed *k* = 0 at different temperatures (red labels). (Bottom) Heatmap representation of *W* as a function of *β* and *γ* for *k* = 0. The inner dark-red region denotes the parameter space where multipartite entanglement could be detected for *W* < 0. The dashed black line separates the positive and negative regions of 〈*W*〉.

The inset of the top panel of Fig. 4 displays the entanglement witness, 〈*W*〉, as a function of *γ* at different temperatures as indicated by the red, bold numbers which are the dimensionless values of *β*. Similar to the bipartite case, it is shown that multipartite entanglement, i.e., 〈*W*〉 < 0, can be generated at intermediate dissipation strength, *γ* ≈ 5 × 10^−3^, provided the temperature is low enough. As temperature increases, thermal fluctuations destroy the equilibrium multipartite entanglement as the system reduced density matrix approaches a totally mixed state. Turning to the main graph in the top panel of Fig. 4, a negative 〈*W*〉 can be seen at low wavenumbers for low temperatures. This implies the existence of multipartite entanglement occurring around *k* ≅ 0 for *β* > 3.

Finally, the lower heatmap of Fig. 4 displays 〈*W*〉 as a function of both *β* and *γ*. Dashed black line separates two distinct regimes of multipartite entanglement. It seems worthwhile mentioning the generic u-shape of the solid lines, indicating that multipartite entanglement develops only within a range of *γ* values and below a critical temperature value. While the latter feature, the existence of a critical temperature is expected, the strong dependence on *γ* is –we believe– novel. The steep slope of the solid curve in terms of *γ* suggests that weak coupling to the bath cannot efficiently relay entanglement and the qubits evolve in time more or less independently of each other. On the other hand, strong coupling with the bath connects the qubit with numerous phonon modes, leading to decoherence and the inhibition of any transmission of entanglement between the qubits.

From here onwards we focus on multilevel systems. One may extend the results above to higher spin values by using appropriate spin operators as the formalism has already been presented in this general form. For example, the form of the Hamiltonian for a spin-1 system (qutrits) is similar to that of the spin-$\frac{1}{2}$ system, with the main difference being the occurrence of the anisotropic term, $\sim {s}_{z}^{2}$ in ${\tilde{H}}_{{\rm{S}}}$. This term contributes only a constant in the case of ${s}_{z}=\frac{1}{2}{\sigma }_{z}$. The polaron transformation can still be defined in this higher dimensional space. In order to apply the transform to *H*_tot_, the Baker-Campbell-Hausdorff formula^46^ is needed, but this does not pose any serious limitation on the calculation.

The bottom panel of Fig. 3 compares EoF_lb_ for bipartite qudit systems from $s=\frac{1}{2}$ up to *s* = 3 in steps of $\frac{1}{2}$ (red bold labels). Notice the log-log scale of the axes. Increasing the dimensionality of a subsystem manifests itself in more brittle entanglement such that at the same noise-level in the bath lower entanglement can be achieved. Therefore the maximal entanglement shift towards weaker interactions by increasing spin dimensions.

**Figure 5:**
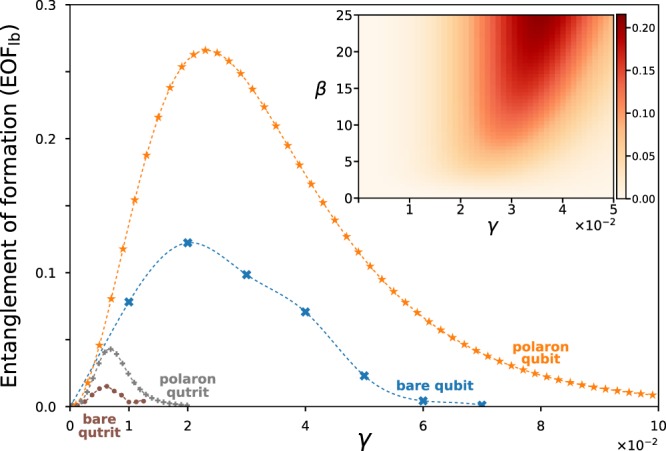
Figure focuses on the case of *s* = 1 and compares the two entanglement measures for two qubits and two qutrits. Both their bare and polaron-dressed measures are given. It is apparent that entanglement is reduced significantly for higher spins. In the inset the lower bound of entanglement of formation (EoF_lb_) is plotted for two polaron qutrits as a heatmap over a range of system-bath coupling, *γ*, and inverse temperature, *β*.

Now looking at Fig. 5, we see that the entanglement of formation exhibits a stochastic resonance behaviour in qutrit systems similar to that in the qubit system, but to a more limited extent.

Moreover, by comparing the curves of qubits and qutrits in the main panel of Fig. 5, we may conclude that observable entanglement in qutrits system can occur only at lower temperatures than in qubit systems. This is expected as the temperature is a proxy for the noise in the phonon-bath. Thus increasing temperature rises noise-levels which destroy correlation. The stochastic resonance thus happens at lower noise levels for the qutrit systems. In summary, the maximal entanglement in both cases depends on temperature and happens at moderate system-bath coupling strengths.

Concluding, we have demonstrated that steady state multipartite and multilevel entanglement can be induced solely through interactions with a common reservoir. The multipartite and multilevel entanglement are experimentally accessible and we propose that an analysis of the system’s structure factors could be used for their detection. Structure factors, both static and dynamic, are commonly used in solid-state physics, and are suited to the capture of correlational structure in few-body systems. They are measurable in scattering experiments, e.g., via neutron scattering in condensed-matter systems, or via light scattering off optical lattices.

We reiterate that the analysis presented here focuses on a bath with a super-Ohmic spectrum for analytical treatment of the polaron perturbation technique. Numerical results with other spectral densities (Ohmic and Lorentzian) show that our conclusions are essentially independent of the precise form of spectral density and applicable to a wide range of systems.

It is clear that the spin-boson models are an important tool in understanding imperfections in quantum gate operation. The results presented above make some first steps in characterising quantitatively steady-state multipartite and multi-dimensional entanglement, thereby helping provide an additional resource for quantum information applications. We envision that entanglement measures based on the structure factor could become a good tool to probe and quantify multipartite qudit entanglement and simultaneously test setups with controllable dissipation, e.g., trapped ions and superconducting qubits.

Fermions (qudits) in optical lattices seem particularly suitable candidates for investigation as these systems are highly controllable, e.g., the potential depth and external magnetic field can control *ε* and Δ, and how strongly these atoms interact with each others. If a thermal cloud of atoms (the bath) were trapped in a different potential and brought in contact with the strongly trapped atoms in the lattice, their interaction, *γ*, could also be tuned. Then one may potentially interrogate the trapped atoms via scattering^28,47^, and deduce the structure factor to quantify entanglement.

## Supplementary information

Supplementary document

## Data Availability

The datasets generated during and/or analysed during the current study are available from the corresponding author on reasonable request.
