# Supplementary material for: Environment mediated multipartite and multidimensional entanglement
Source: Sci Rep. 2019 Jun 24;9:9147. doi: 10.1038/s41598-019-45496-2 (PMC6591356; doi:10.1038/s41598-019-45496-2)
Supplement: Supplementary file 1 — Supplementary document [file 41598_2019_45496_MOESM1_ESM.pdf]

Environment mediated multipartite and multidimensional entanglement

Chee Kong Lee,[1,2] Mojdeh S. Najafabadi,[3] Daniel Schumayer,[3]
Leong Chuan Kwek,[1,4,5,6] and David A. W. Hutchinson[1,3,7,*]

[1] *Centre for Quantum Technologies, National University of Singapore, 117543, Singapore*
[2] *Department of Chemistry, Massachusetts Institute of Technology, Cambridge, Massachusetts 02139, USA*
[3] *Dodd-Walls Centre for Photonic and Quantum Technologies, Department of Physics, University of Otago, Dunedin, New Zealand*
[4] *Institute of Advanced Studies, Nanyang Technological University, 60 Nanyang View Singapore 639673, Singapore*
[5] *National Institute of Education, 1 Nanyang Walk Singapore 637616, Singapore*
[6] *MajuLab, CNRS-UNS-NUS-NTU International Joint Research Unit, UMI 3654, Singapore*
[7] *Department of Physics, Clarendon Laboratory, University of Oxford , Parks Road, Oxford OXI 3PU, United Kingdom*

Appendix A: Details of multipartite qubit system

We now discuss in a little more detail the qubit system. The total Hamiltonian for N qubits, $H_{\rm tot} = H_{\rm S} + H_{\rm B} + H_{\rm I}$, in the open quantum system formalism is given by

$$H_{\rm S} = \frac{1}{2}\sum_{j}^{N}\left[\epsilon\sigma_z^{(j)} + \Delta\sigma_x^{(j)}\right] \tag{1a}$$

$$H_{\rm B} = \sum_{n}\omega_n b_n^\dagger b_n \tag{1b}$$

$$H_{\rm I} = \sum_{nj} g_n\sigma_z^{(j)}\left(b_n^\dagger + b_n\right), \tag{1c}$$

where $H_{\rm S}$, $H_{\rm B}$ and $H_{\rm I}$ are the system, bath and the system-bath coupling Hamiltonians, respectively. The system is described by a group of N non-interacting identical two-level systems with energy splitting ϵ and tunnelling matrix element Δ. The corresponding polaron transformation is

$$P = \sum_j \sigma_z^{(j)}\sum_n \frac{g_n}{\omega_n}(b_n^\dagger - b_n). \tag{2}$$

Up to a constant we obtain $\widetilde{H}_{\rm tot} = \widetilde{H}_{\rm S} + \widetilde{H}_{\rm B} + \widetilde{H}_{\rm I}$ with

$$\widetilde{H}_{\rm S} = \sum_j\left[\frac{\epsilon}{2}\sigma_z^{(j)} + \frac{\Delta_R}{2}\sigma_x^{(j)}\right] - \sum_n\frac{g_n^2}{\omega_n}\sum_{jk}\sigma_z^{(j)}\sigma_z^{(k)} \tag{3a}$$

$$\widetilde{H}_{\rm B} = H_{\rm B} \tag{3b}$$

$$\widetilde{H}_{\rm I} = \sum_j\left[\sigma_x^{(j)}V_x + \sigma_y^{(j)}V_y\right]. \tag{3c}$$

For $\widetilde{H}_{\rm S}$, the tunnelling element in $\widetilde{H}_{\rm S}$ is renormalized due to the system bath coupling,

$$\Delta_{\rm R} = \Delta\exp\left(-\frac{2}{\pi}\int_0^\infty\frac{J(\omega)}{\omega^2}\coth\left(\frac{\beta\omega}{2}\right)d\omega\right). \tag{4}$$

In order to get some insight into the behaviour of the renormalised tunnelling rate, $\Delta_{\rm R}$, one may evaluate this integral for a generic super-Ohmic spectral density

$$J(\omega) = \gamma\left(\frac{\omega}{\omega_{\rm c}}\right)^k e^{-\omega/\omega_{\rm c}} \tag{5}$$

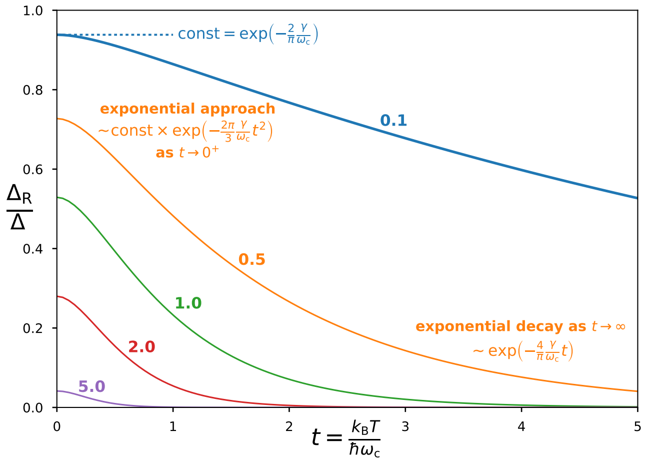

FIG. 1. (Colour online) The dimensionless ratio of $\Delta_{\rm R}/\Delta$ is plotted as a function of dimensionless temperature, $t = k_{\rm B}T/(\hbar\omega_{\rm c})$. The bold values close to each curves give the actual value of the coupling strength, γ. Asymptotic behaviour both at low- and high-temperatures are indicated. The $t\to 0^+$ limit also demonstrates that increasing γ reduced the ratio exponentially fast, i.e, the tunnelling term in the polaron picture, $\Delta_{\rm R}$, quickly diminishes with stronger coupling.

where k has to be bigger that 2 to mollify the singularity at $\omega = 0$. Substituting this expression into the integral one may arrive at

$$\ln\left(\frac{\Delta_{\rm R}}{\Delta}\right) = -\frac{2}{\pi}\left(\frac{\gamma}{\omega_c}\right)\Gamma(k-1)\left[2t^{k-1}\zeta(k-1,t)-1\right] \tag{6}$$

where $\zeta(s,a)$ is the generalised Riemann-zeta function and $t = k_{\rm B}T/(\hbar\omega_{\rm c})$ is a dimensionless temperature. Let us choose $k = 3$ in order to be consistent with the spectral density used in the main body of the work and examine the low- and high-temperature asymptotic of this expression. As $t\to 0^+$ the generalised Riemann-zeta function develops a singularity, however, its pre-factor t^{k-1} cancels that precisely and we obtain

$$\frac{\Delta_{\rm R}}{\Delta} \cong \exp\left(-\frac{2}{\pi}\left(\frac{\gamma}{\omega_{\rm c}}\right)\left[1+\frac{\pi^2}{3}t^2\right]\right) \to \exp\left(-\frac{2}{\pi}\frac{\gamma}{\omega_{\rm c}}\right). \tag{7}$$

Thus the renormalised tunnelling term approaches a constant which depends on the coupling strength and cut-off frequency. This result demonstrates that with increasing system-bath coupling the tunnelling term vanishes exponentially. In the opposite, high-temperature limit, the zeta function vanishes as $(k-2)t^{2-k}+2t^{1-k}$, thus in our case $(\gamma > 0)$

$$\frac{\Delta_{\mathrm{R}}}{\Delta} \cong \exp\left(-\frac{4}{\pi}\frac{\gamma}{\omega_{\mathrm{c}}}t\right) \to 0. \tag{8}$$

As anticipated, for decoupled systems, i.e., $\gamma = 0$, the tunnelling term is not affected by the bath in any way, thus $\Delta_{\mathrm{R}} = \Delta$.

After analysing the renormalised tunnelling term in detail, let us return to the formalism used for the two qubit case. As for the general spin case, here the system-bath coupling also induces a qubit-qubit interaction term, $\sigma_z^{(j)}\sigma_z^{(k)}$, which appears in $\widetilde{H}_{\mathrm{S}}$. The system bath coupling in the polaron picture, $\widetilde{H}_{\mathrm{I}}$, assumes a form very different from H_{I}. The bath operators entering into the system-bath coupling are

$$V_x = \frac{1}{4}\Delta\left(D_+^2 + D_-^2 - 2B\right) \tag{9a}$$

$$V_y = -\frac{i}{4}\Delta\left(D_-^2 - D_+^2\right), \tag{9b}$$

with $D_\pm = \exp\left(\pm\sum_n \frac{g_n}{\omega_n}\left(b_n^\dagger - b_n\right)\right)$ and $\langle D_\pm^2\rangle_{\mathrm{B}} = R$. The bath correlation functions, $C_{\ell\ell'}(\tau) = \langle V_\ell(\tau)V_{\ell'}\rangle_{\mathrm{B}}$ are given by

$$C_{xx}(\tau) = \frac{1}{4}\Delta_R^2\left[\cosh\left(\phi(\tau)\right) - 1\right] \tag{10a}$$

$$C_{yy}(\tau) = \frac{1}{4}\,\Delta_R^2 \sinh\left(\phi(\tau)\right), \tag{10b}$$

where

$$\phi(\tau) = \frac{4}{\pi}\int_0^\infty \frac{J(\omega)}{\omega^2}\frac{\cosh\left(\frac{1}{2}\left(\beta - 2\tau\right)\omega\right)}{\sinh\left(\frac{1}{2}\beta\omega\right)}\,d\omega. \tag{11}$$

The diagonal correlation function, $C_{zz}(\tau)$, together with cross-correlations, e.g., $C_{xz}(\tau)$, vanish identically using the full polaron transformation described in the text.

Appendix B: Ohmic and Lorentzian baths Here we study the equilibrium entanglement induced by a common Ohmic or Lorentzian bath. These baths are represented by the spectral densities

$$J_{\mathrm{Ohmic}}(\omega) = \gamma\,\omega\,\mathrm{e}^{-\omega/\omega_c} \tag{12a}$$

$$J_{\mathrm{Lorentz}}(\omega) = \gamma\,\frac{\omega\,\omega_c}{\omega^2 + \omega_c^2}, \tag{12b}$$

respectively. Both of these densities behave as $J(\omega) \propto \omega$ for $\omega \ll 1$, thus an essential singularity appears at $\omega = 0$ in the expressions for Δ_{R} and $\phi(\tau)$. Consequently, the polaron method used in the main text is not applicable for these baths as it suffers from nonphysical divergences [1, 2]. The integrals in Δ_{R} and $\phi(\tau)$ are all divergent for any non-zero coupling strengths, thus the tunnelling element, Δ_{R}, is always normalised to zero. Therefore, here we only present the numerical results from the imaginary time path integral simulations.

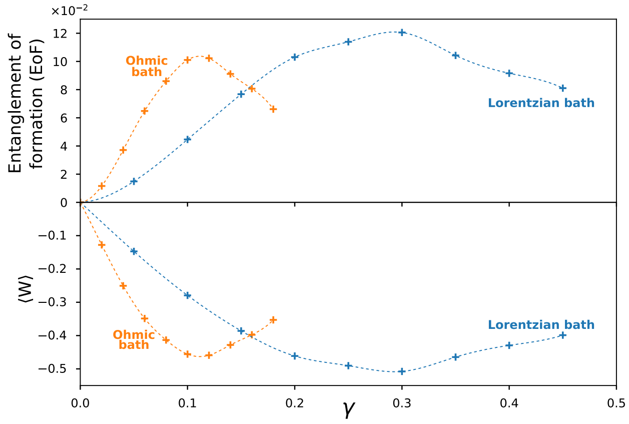

FIG. 2. (Colour online) Entanglement measures, EoF and $\langle W\rangle$, are plotted as functions of the coupling strength, γ, for two qubits coupled to either an Ohmic (orange) or Lorentzian bath (blue). In both cases the inverse temperature is $\beta = 10$, while the cut-off angular frequencies were $\omega_{\mathrm{c}} = 8$ for the Ohmic and $\omega_{\mathrm{c}} = 5$ for the Lorentzian bath. Note that the values of the ordinate in the top figure need to be multiplied by $\times 10^{-2}$.

In the spin-boson models, e.g., the one used in this work, where the bath is harmonic, the trace over the bath degrees of freedom for ρ_{S} can be performed analytically and –within the path integral formalism– we arrive at the Feynman-Vernon influence functional [1, 3]. Employing the Hubbard-Stratonovich transformation and realising that the influence functional can be unravelled by an auxiliary stochastic field, $\xi(\tau)$, leads to a time-dependent Hamiltonian $H_{\mathrm{st}} \sim \frac{1}{2}\left[\epsilon\sigma_z + \Delta\sigma_x\right] + \xi(\tau)\sigma_z$ governing the imaginary time evolution. All of the effects of the bath are accounted for by the coloured noise term, $\xi(\tau)$. The trace over the bath now corresponds to averaging the imaginary time dynamics over realisations of the noise. The primary benefit of this path integral approach is that it provides the entire reduced density matrix from a single Monte Carlo calculation and arbitrary spectral density, $J(\omega)$, may be used. In our calculations, $10^8 - 10^{11}$ samples achieved convergence. Further details of the numerical implementation can be found in Refs. [4, 5]. Due to the high computational cost of this method, we only study the two qubit case $(N = 2)$ for the weak and intermediate system-bath coupling regimes. In the following, we use $\beta = 10$, $\epsilon = 0$ and $\Delta = 1$. The entanglement of formation and structure factor entanglement witness between two non-interacting qubits in a common Ohmic bath and the same quantities for a Lorentzian bath are shown in Fig. 2. It can be seen that the features in both cases are qualitatively similar

to those obtained using a super-Ohmic spectral density in the main text. Therefore, the general observations made in our main text should not be sensitive to the spectral density of the bath.

Appendix C: Scaling peak of entanglement and structure factor Finally, we present an approximate power law scaling relation in the qudit systems. Fig. 3 shows the scaling of peak entanglement for high dimensional bipartite and multipartite systems. The $\mathrm{EoF_{lb}}$ peaks obey a consistent power law, although some fluctuation is seen in the peak of the structure-factor-based entanglement measure for multipartite system. Relations vary between approximately linear and a square root behaviour. An analytic treatment remains the subject of further work.

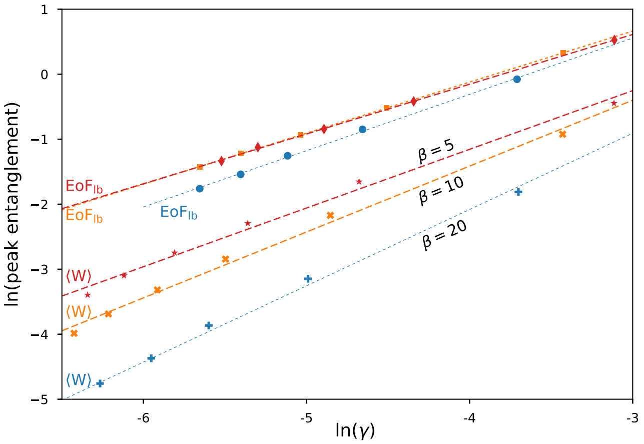

FIG. 3. (Colour online) The value of peak entanglement measures, $\mathrm{EoF_{lb}}$ and $\langle W \rangle$, as a function of interaction strength, γ, as a log-log plot together with linear fits. Powers vary between approximately 0.5 and 1.0. The inverse temperature values, $\beta = 5$, 10 and 20, are indicated as appropriate.

* david.hutchinson@otago.ac.nz

[1] U. Weiss, *Quantum dissipative systems*, Vol. 13 (World scientific, 2012).
[2] W. Zwerger, Zeitschrift für Physik B Condensed Matter **53**, 53 (1983).
[3] R. P. Feynman and F. L. Vernon, Jr., Annals of Physics **24**, 118 (1963).
[4] C. K. Lee, J. Moix, and J. Cao, The Journal of Chemical Physics **136**, 204120 (2012).
[5] J. M. Moix, Y. Zhao, and J. Cao, Physical Review B **85**, 115412 (2012).
